# Supplementary material for: Individual subunits of a rhinovirus causing common cold exhibit largely different protein-RNA contact site conformations
Source: Commun Biol. 2020 Sep 29;3:537. doi: 10.1038/s42003-020-01269-6 (PMC7525237; doi:10.1038/s42003-020-01269-6)
Supplement: Supplementary file 1 — Supplementary Information [file 42003_2020_1269_MOESM1_ESM.pdf]

SUPPLEMENTARY INFORMATION TO:

Individual subunits of a rhinovirus causing common cold exhibit largely different protein-RNA contact site conformations

Dieter Blaas

Max Perutz Laboratories, Center of Medical Biochemistry, Medical University of Vienna, A-1030 Vienna, Austria

Email: dieter.blaas@meduniwien.ac.at

This PDF file includes: Supplementary Figures 1 to 6

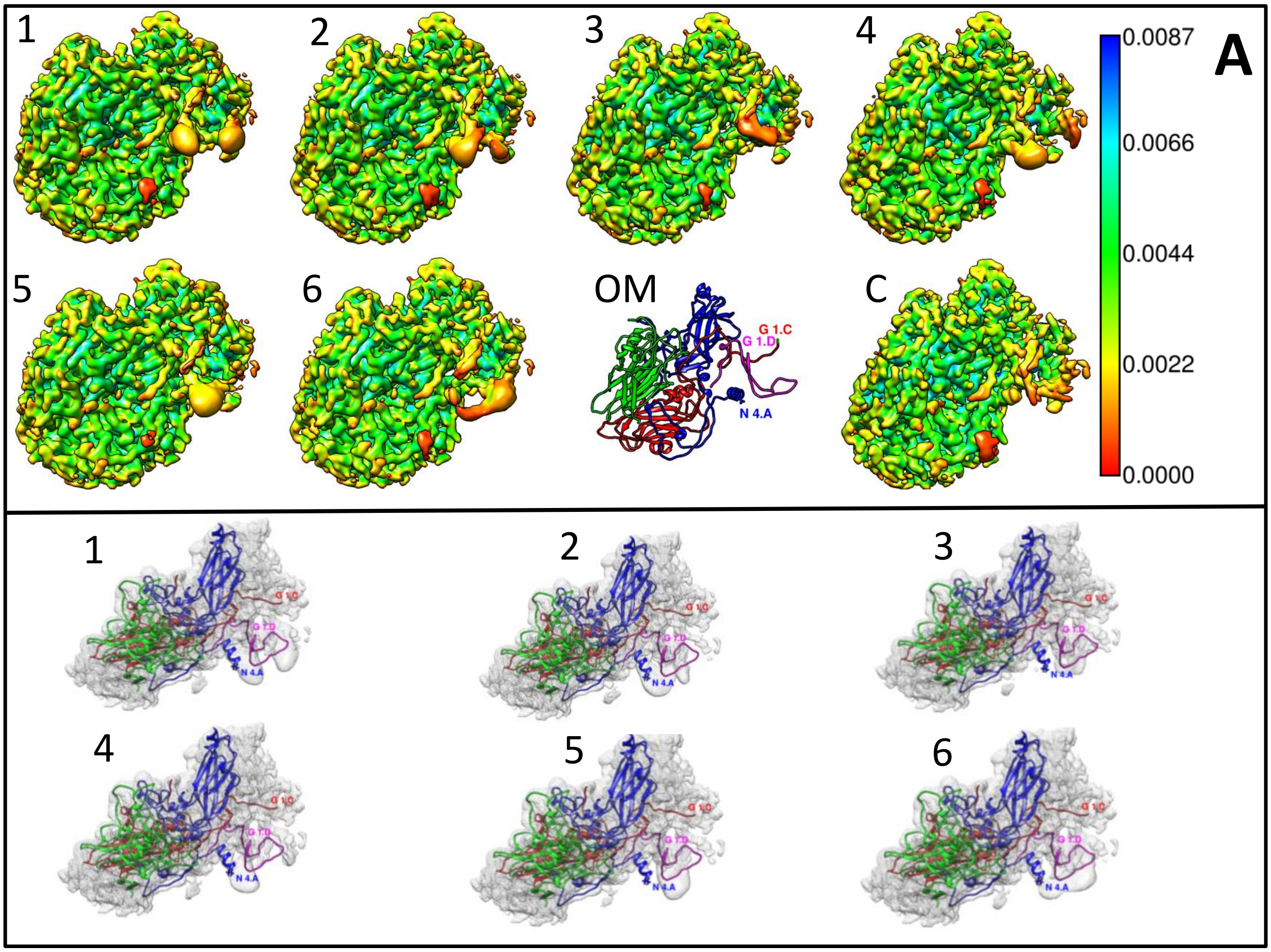

Supplementary Figure 1A: Three-dimensional classification without alignment of single asymmetric units of RV-A89 into six classes (1 to 6) from the icosahedrally expanded cryo-EM dataset of RV-A89. The model of an asymmetric unit (ribbon diagram in Fig. 1B) was used to excise a map from the entire viral volume (Fig. 1A) and used as a reference and for calculating a mask (Fig. 1C, D, E, blue). As a control, the identical classification procedure was carried out with the number of 3D-classes set to one ('C'; same map as in Fig. 2D). Upper panel, orientation of the view is onto the N-terminal helix of VP1 as also seen in the original model (OM). Rendered with Chimera at sigma=10 and coloured according to 'volume data gradient norm' as indicated with the colour key bar. Lower panel, same maps as above but rendered as mesh overlaying the atomic model shown as ribbon diagram. VP1, blue; VP2, green; VP3, red; VP4, magenta. Amino acid residues at or close to the N-termini of the VPs are indicated as N4.A, VP1; G1.C, VP3; and G1D, VP4. Viewed sidewise for a better appreciation of the densities corresponding to the above N-terminal extension of VP1.

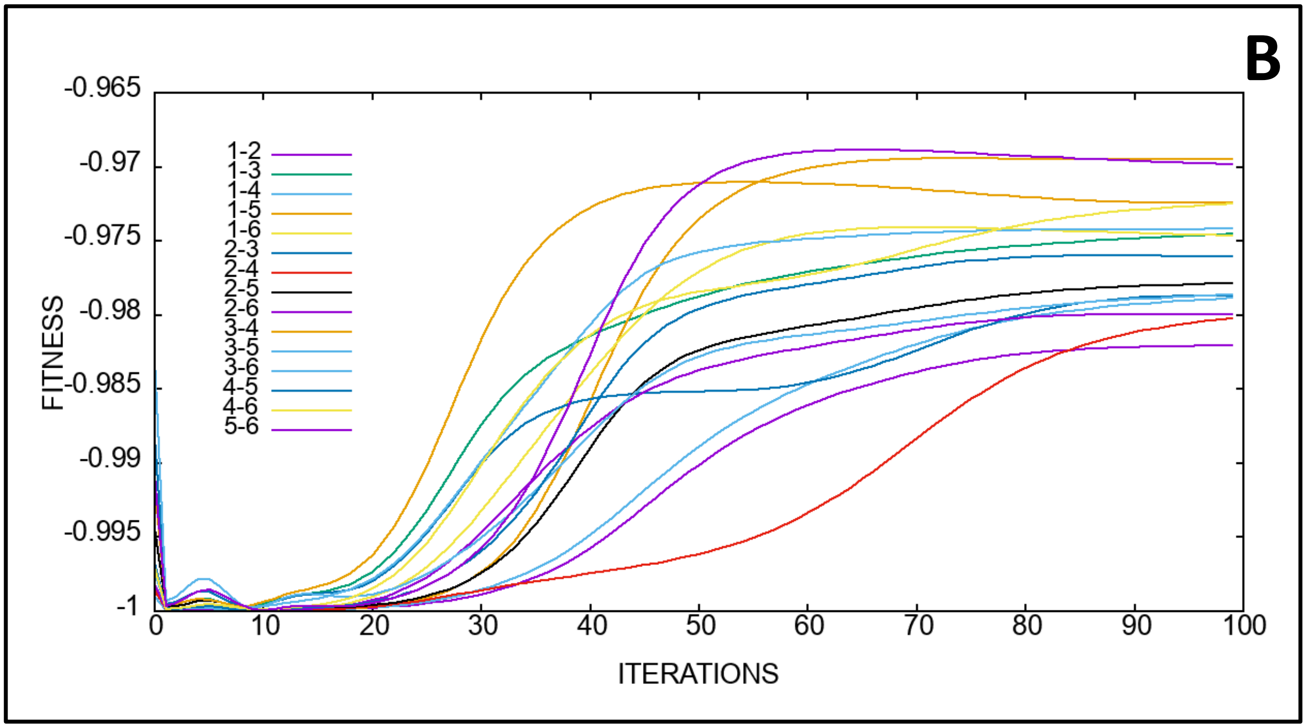

Supplementary Figure 1B: Evolution of fitness of the 15 pairs of six 3D-classes of single asymmetric units as function of the number of iterations. Note that the lowest similarity, i.e. the highest fitness value, is found for the pairs of class 1 and 5 and of class 5 and 6.

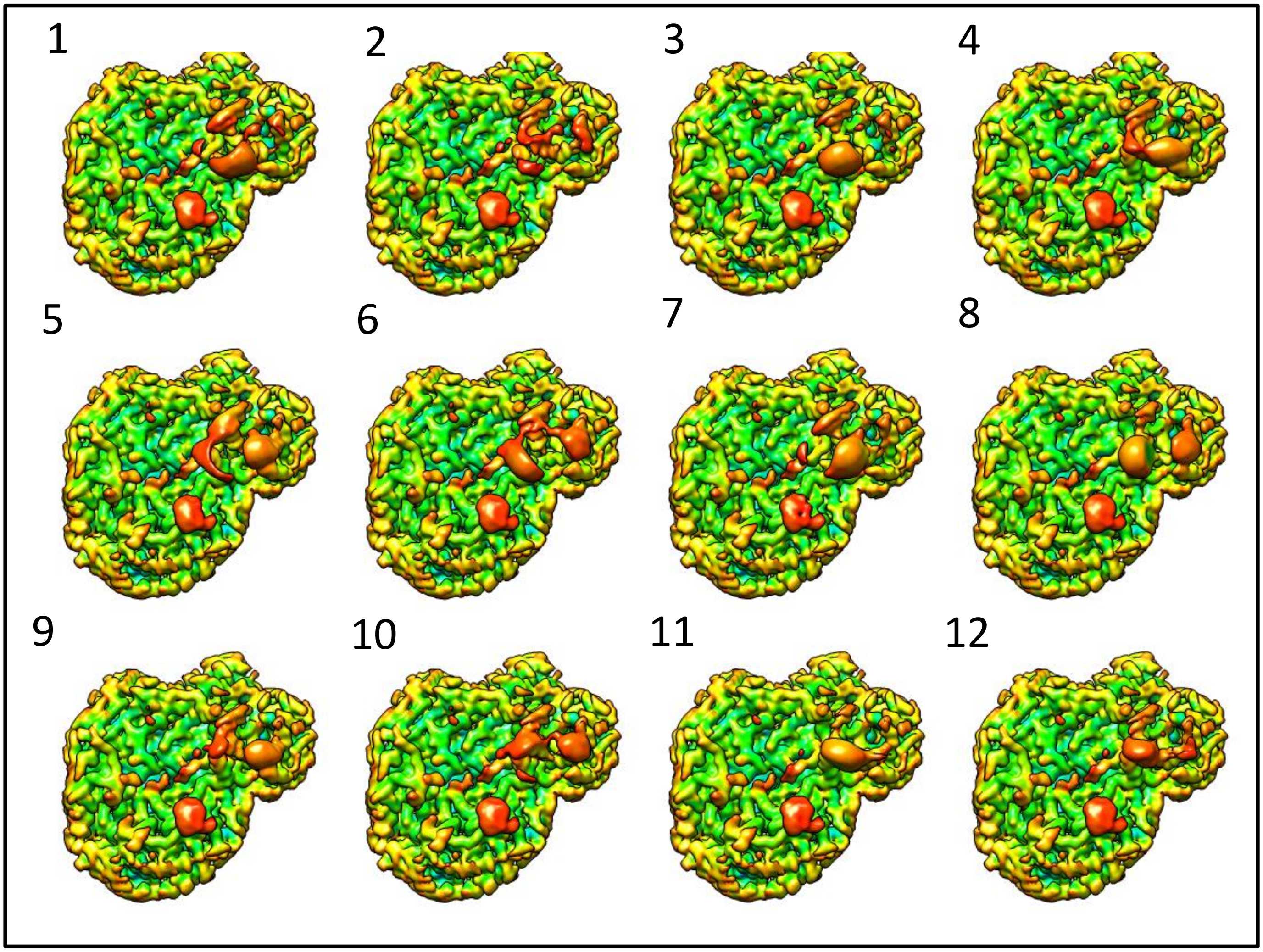

Supplementary Figure 2: Results of 3D classification of single asymmetric units into 12 3D-classes. For methods and corresponding colour key bar see Supplementary Figure 1A.

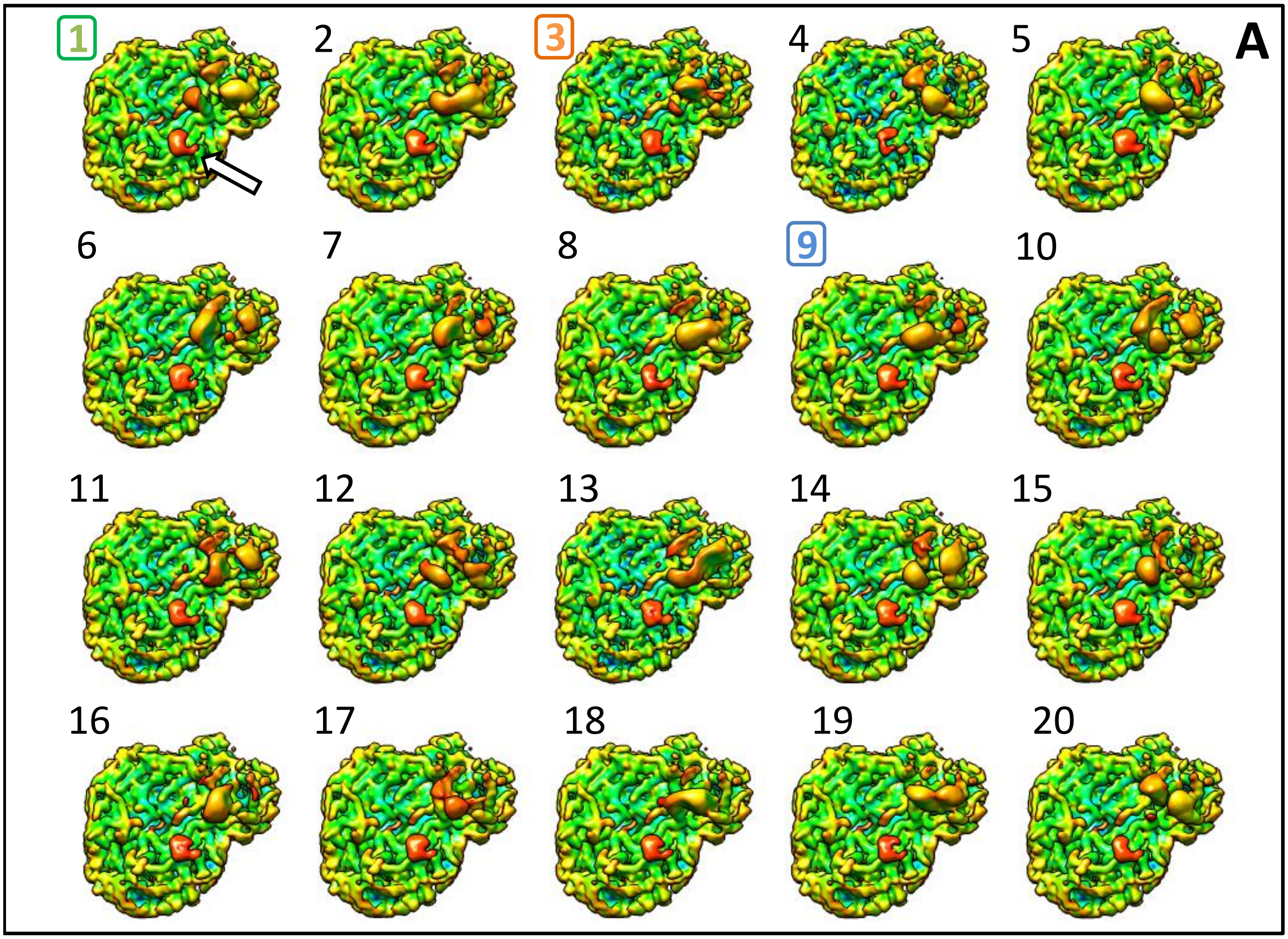

Supplementary Figure 3A: Results of 3D classification of single asymmetric units into twenty 3D-classes. For methods and colour key bar see Supplementary Figure 1A. Boxed numbers refer to 1) the class most often present only once per particle image (see Supplementary Figure 3B), 3) the class differing most from all other classes (see Supplementary Figure 3C), and, 9) the class represented most frequently per particle image (i.e. 5 times; see Supplementary Figure 3B). Arrow points to density present in all classes with very minor shape differences.

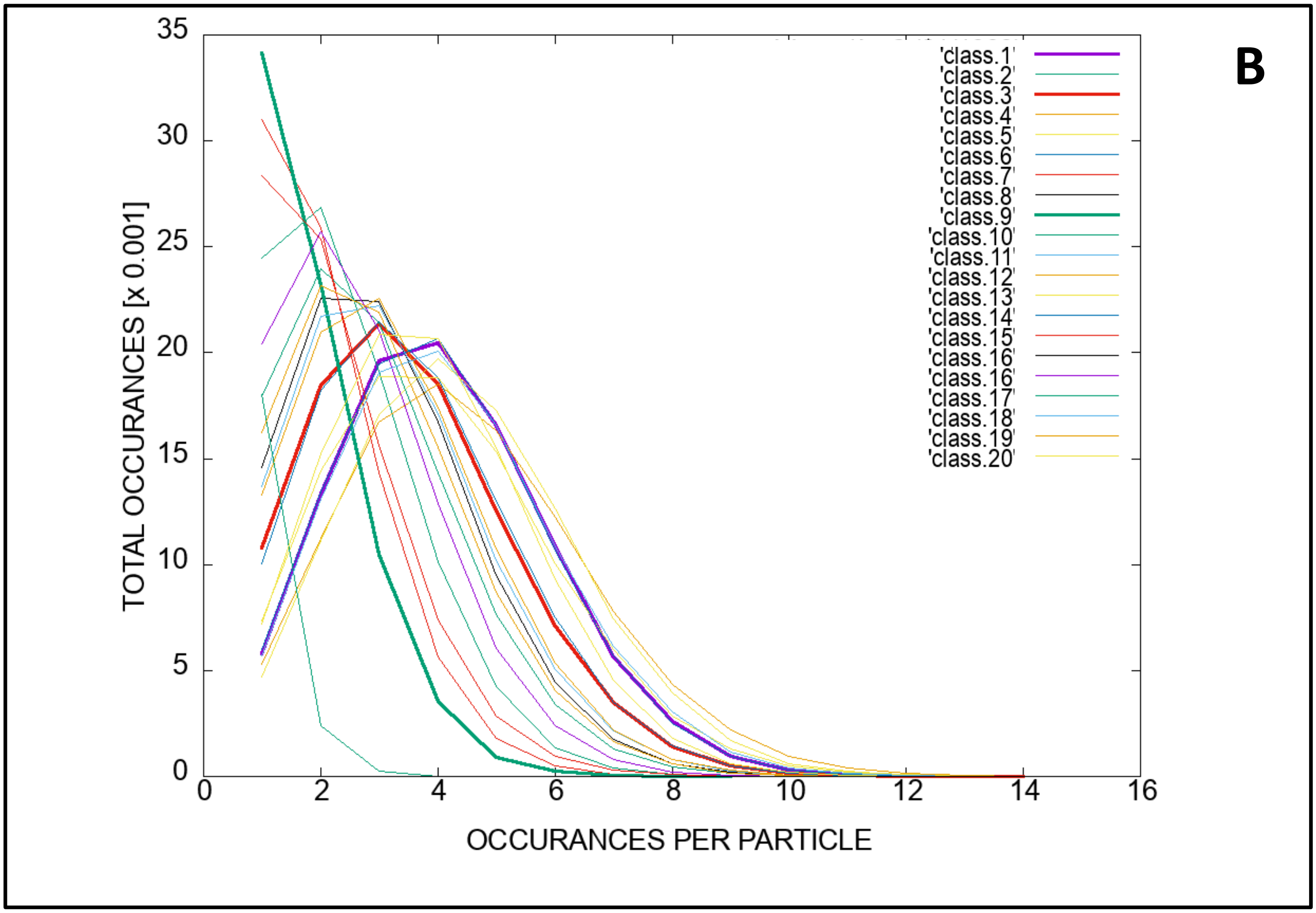

Supplementary Figure 3B: Number of occurrences of a given 3D-class per particle. Note that none of them occurred more than 14 times per particle. Thick lines correspond to those classes chosen for reconstruction of the entire virion maps on the basis of the angular orientations of the particle images within the respective 3D-class shown in Fig. 4. Note that class 1 has the highest representation per particle image (i.e. peak at 5 occurrences), class 3 is most different (highest fitness value) from all other classes (see Fig. 3C), and class 9 has the lowest representation per particle image (i.e. peak at 1 occurrence).

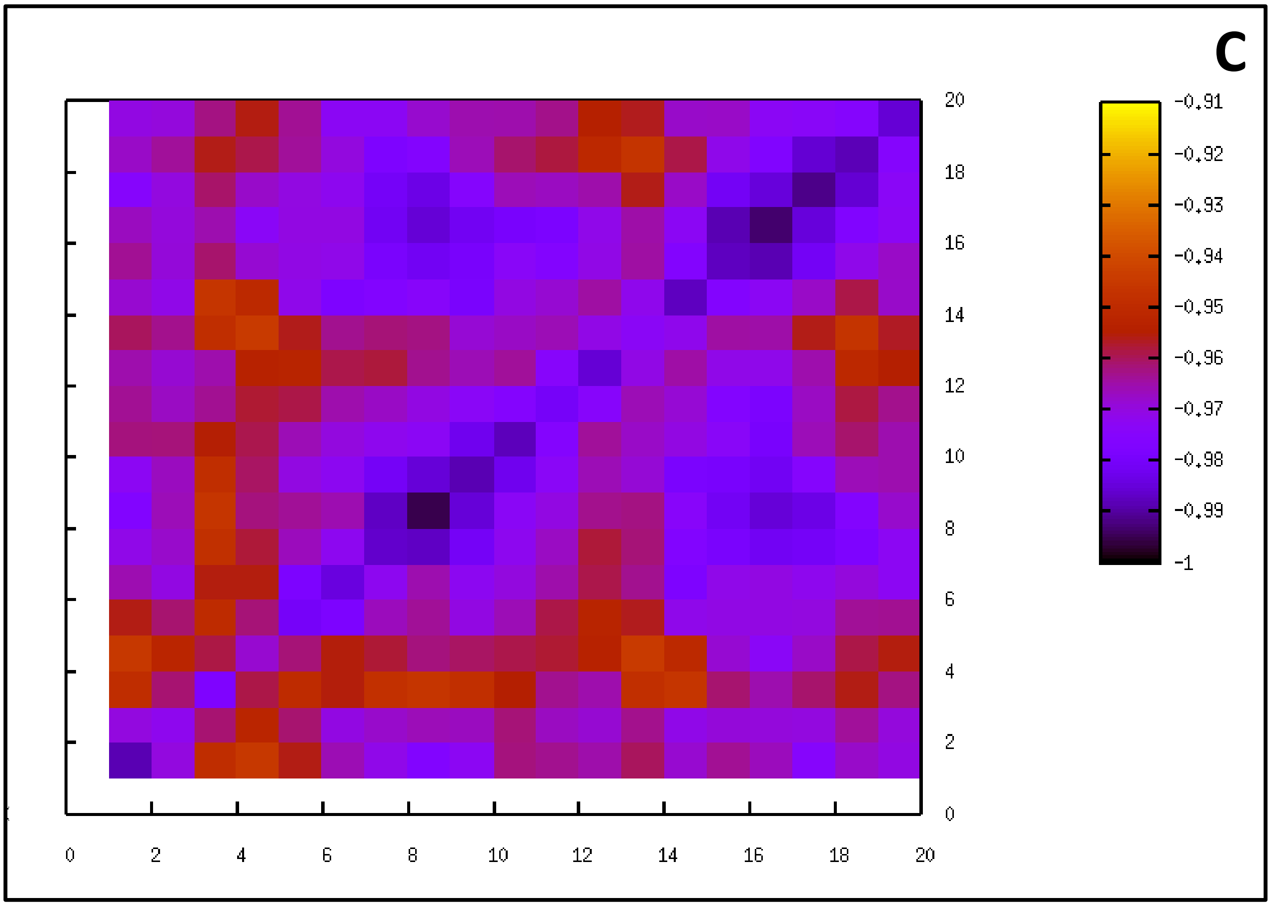

Supplementary Figure 3C: Heat map displaying the fitness of all 400 pairs of the 20 3D-classes. Note the mostly dark blue or black diagonal indicating the base level of the fitness value for identical maps and the accumulation of red fields indicating a high dissimilarity between the 3D-map of class 3 and the 3D-maps of all other classes.

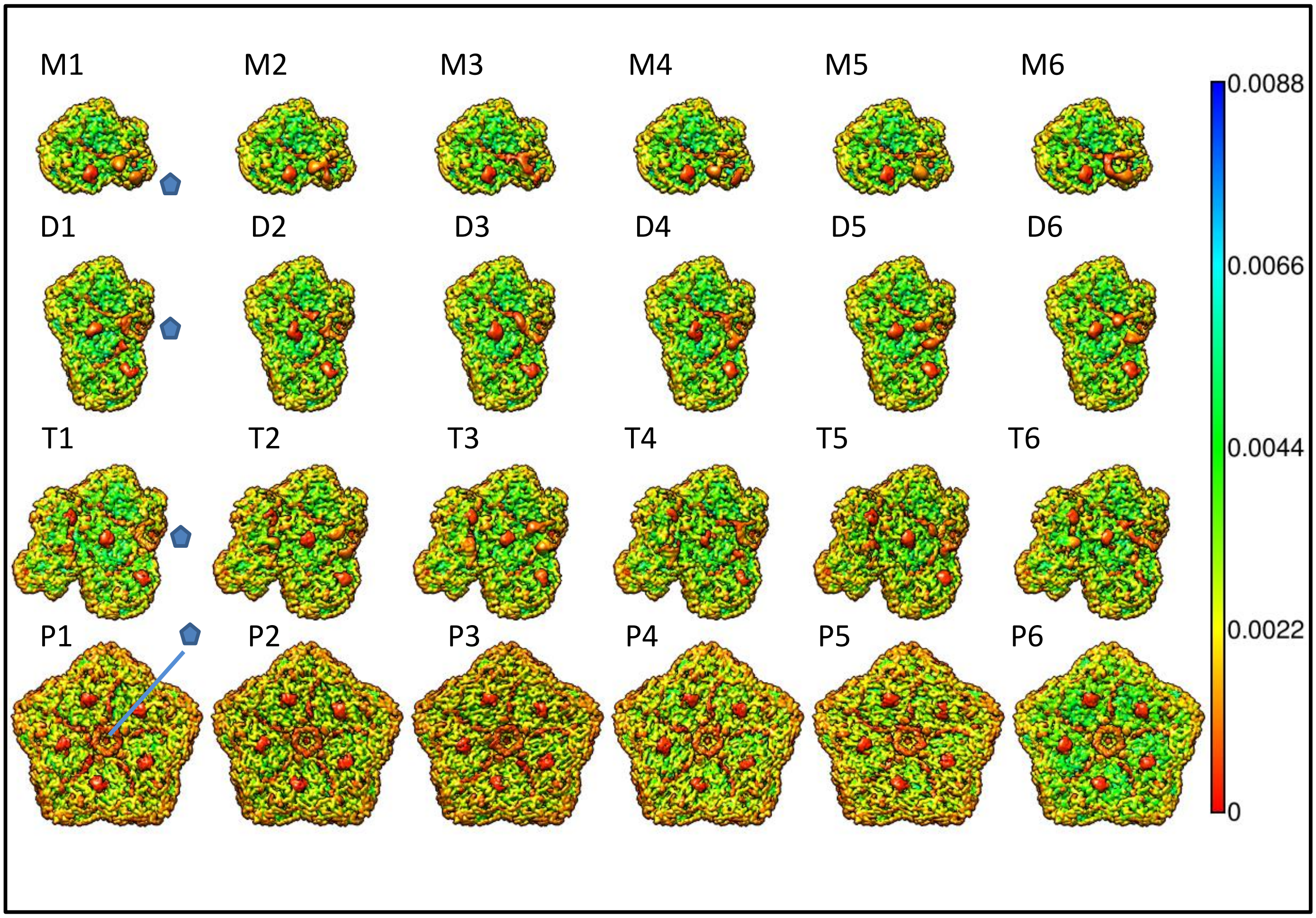

Supplementary Figure 4: Three-dimensional classification, without alignment, into six classes of monomers (M), dimers (D), trimers (T), and pentamers (P) of asymmetric units of RV-A89. For the respective masks see Supplementary Figures 1C,D,E. A five-fold axis of symmetry is left from the blue pentagon within the density (M1,D1,T1) and where indicated with the line (P1). For better comparison all subunits are displayed as seen from inside the virion along a five-fold (z) axis (best seen for the pentamer). Rendered with Chimera at sigma=10 and coloured according to 'volume data gradient norm' as indicated by the colour bar.

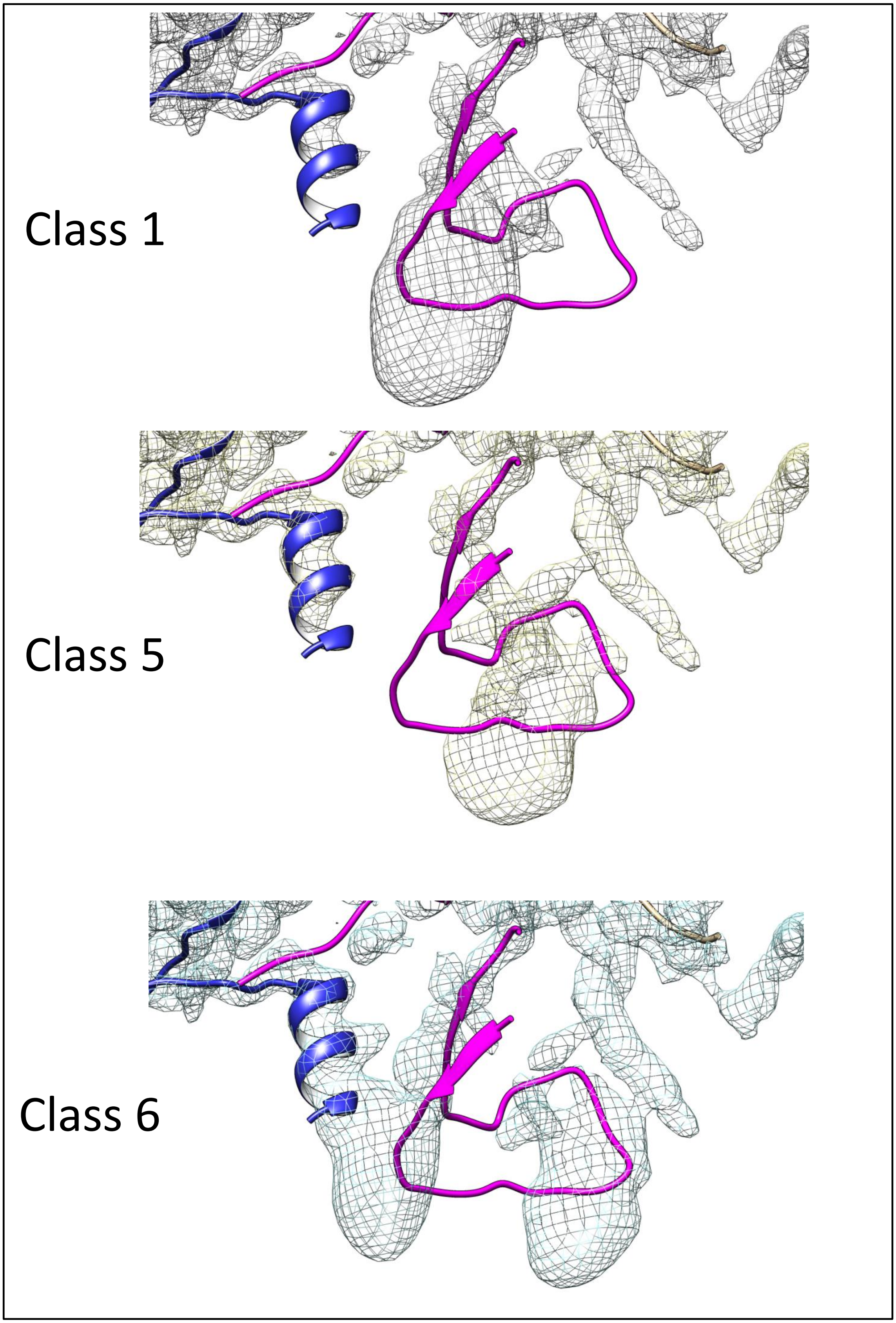
Class 1
Class 5
Class 6

Supplementary Figure 5: Zoom-in onto the most diverging densities of class1, class5, and class6 from the classification into six 3D classes (for the entire subunits see Supplementary Figure 1). The backbone coordinates of the original fit (pdb-6SK7), with the gaps filled with the backbone coordinates from the more complete model of the X-ray structure of RV-A16 (1AYN) are shown for VP1, blue and VP4, magenta. Note that the differences in position can be roughly estimated from the densities to possibly lie in a range of 2 to 3 Å.

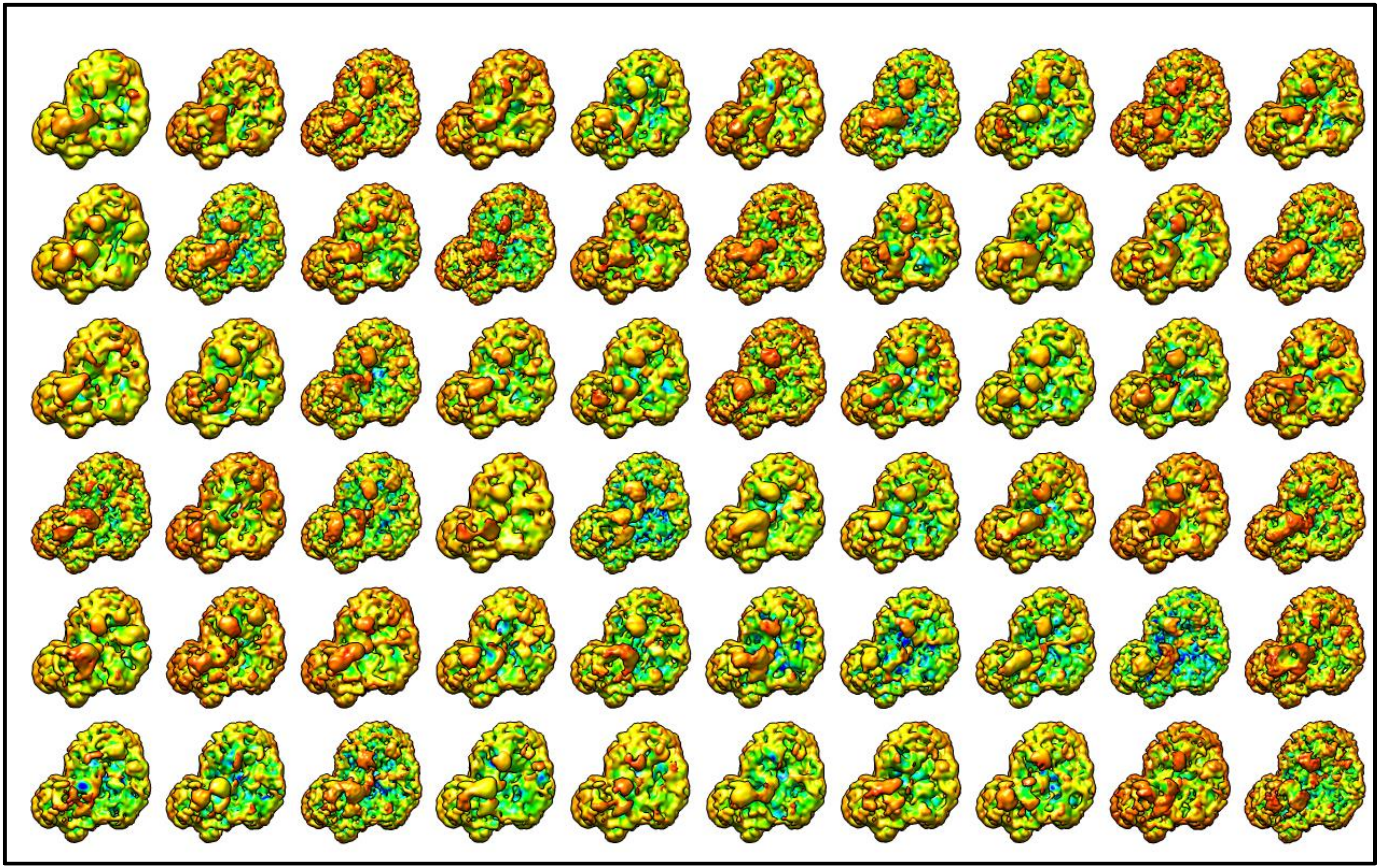

Supplementary Figure 6: Three-dimensional classification, without alignment, into sixty classes of asymmetric units of RV-A89 of the icosahedrally expanded dataset after signal subtraction (i.e. ‘Particle subtraction’ in Relion-3.1-beta). Mask and reference volume were as in the classification depicted in Figures 2 and 3 but the box was recentred and reduced to 150 x 150 x 150 px (instead of 450 x 450 x 450 px). Rendered with Chimera as in Supplementary Figure 3A. Note that the resolution differs from class to class because of the reduced number of particles per class resulting from the sixty classes. Note that the class distribution, i.e. the fraction of particle images segregating into the various classes, was between 0.00961 and 0.0336.
